# Draft genome assembly of *Lactobacillus johnsonii* UMB3423 from a voided urine sample

**DOI:** 10.1128/mra.00333-24

**Published:** 2024-06-04

**Authors:** Natalie Stegman, Maria Steiling, Briana Jackson, Cerena Sedano, Catherine Putonti

**Affiliations:** 1 Bioinformatics Program, Loyola University Chicago, Chicago, Illinois, USA; 2 Department of Biology, Loyola University Chicago, Chicago, Illinois, USA; Wellesley College, Wellesley, Massachusetts, USA

**Keywords:** *Lactobacillus johnsonii*, urinary tract, urobiome

## Abstract

While the urogenital microbiota of asymptomatic females is often dominated by species of *Lactobacillus*, *Lactobacillus johnsonii* is not a common member. It is more frequently found in the gastrointestinal tract. Here, we present the draft genome sequence of *L. johnsonii* UMB3423, which was isolated from a voided urine sample.

## ANNOUNCEMENT

*Lactobacillus crispatus*, *Lactobacillus gasseri*, *Lactobacillus iners*, and *Lactobacillus jensenii* are frequent dominant members of the asymptomatic (“healthy”) female urogenital microbiota (1–3). The species *Lactobacillus johnsonii* also has been identified in voided urine (4) and vaginal samples (5, 6). *L. johnsonii*, however, is more frequently found in the human gastrointestinal (GI) tract [see review reference (7)], and strains have been explored for their use as a probiotic for GI and vaginal health (8, 9). Continuing our group’s efforts to characterize bacterial species of the female urinary tract (10), we sequenced a strain of *L. johnsonii* isolated from a voided urine sample from a female with type 2 diabetes.

Strain UMB3423 was isolated from the urine sample using the enhanced quantitative urine culture method (11), which isolates bacteria from urine samples using a variety of culture conditions and volumes of sample, as part of a prior Institutional Review Board (IRB)-approved study (LUC # 204197) in 2016 (12). The strain was identified as *L. johnsonii* by MALDI-TOF, performed as described previously (13), and stored at −80°C in the Loyola Urinary Education and Research Collaborative (LUEREC) collection. We obtained the freezer stock of this isolate from this collection and streaked it onto a deMan-Rogosa-Sharpe (MRS) agar plate and incubated at 35°C with 5% CO_2_ for 48 hours. MRS medium with 1 mL/L of Tween 80 (Sigma-Aldrich) was inoculated with a single colony from the plate and incubated for 48 hours at 35°C with 5% CO_2_. DNA was extracted using the DNeasy blood and tissue kit (Qiagen) following the manufacturer’s protocol for Gram-positive bacteria.

DNA was sent to SeqCenter (Pittsburgh, PA USA) where libraries were prepared using the Illumina DNA Prep kit and custom IDT 10 bp unique dual indices with a target insert size of 320 bp. The library was sequenced on the Illumina NovaSeq 6000 platform, producing 2,220,089 pairs of reads (2 × 151 bp). Raw reads were trimmed using BBDuk (BBMap suite, v39.01; https://sourceforge.net/projects/bbmap/) and assembled using SPAdes v3.15.5 with the --only-assembler parameter (14). Genome coverage was calculated using BBMap’s BBWrap script. The genome assembly was annotated upon submission to NCBI by the Prokaryotic Genome Annotation Pipeline v6.6 (15). Genome comparisons were conducted via BV-BRC (16, 17) and the Type Strain Genome Server (18, 19). Default parameters were used for all software tools unless otherwise noted.

The *L. johnsonii* UMB3423 assembly includes 2,066,246 bp in 37 contigs with a GC content of 34.2% (genome coverage = 290.615×; N_50_ = 113,600 bp). Upon submission of the assembly to NCBI, the average nucleotide identity (ANI) was computed, producing a score of 94.83%, slightly below the 95% threshold commonly used to distinguish between species (20). This prompted us to further investigate *L. johnsonii* UMB3423 and other genomes for the species (see Table 1). Currently only 5—including UMB3423—of the nearly 1,000 publicly available *L. johnsonii* genomes are from female urogenital samples. While both ANI and digital DNA-DNA hybridization (dDDH) suggest that UMB3423 is genetically different from other *L. johnsonii* strains, further sequencing of strains from the female urogenital tract is needed.

**TABLE 1:** Results of comparisons of the *L. johnsonii* UMB3423 genome sequence to publicly available assemblies

Genome comparison tool	Closest genome (accession)	Score
BV-BRC Similar Genome Finder	*L. johnsonii* NCC 533 (AE017198.1)	Mash distance = 0.0468354
Type Strain Genome Server	*L. johnsonii* ATCC 33200 (GCF_000159355.1)	dDDH, d_4_ = 59.0%

## Data Availability

The draft genome sequence was deposited at DDBJ/ENA/GenBank under the accession JAVTXH00000000. Raw reads are available via SRA, accession SRR26087526.
